# Mitochondrial Mg^2+^ homeostasis decides cellular energy metabolism and vulnerability to stress

**DOI:** 10.1038/srep30027

**Published:** 2016-07-26

**Authors:** Ryu Yamanaka, Sho Tabata, Yutaka Shindo, Kohji Hotta, Koji Suzuki, Tomoyoshi Soga, Kotaro Oka

**Affiliations:** 1 Center for Biosciences and Informatics, School of Fundamental Science and Technology Graduate School of Science and Technology, Keio University, Yokohama, 223-8522, Kanagawa, Japan; 2 Institute for Advanced Biosciences, Keio University, Kakuganji, Tsuruoka, Yamagata, 997-0052, Japan; 3 Center for Science and Technology for Designing Functions, School of Integrated Design Engineering, Graduate School of Science and Technology, Keio University, Yokohama, Kanagawa, 223-8522, Japan

## Abstract

Cellular energy production processes are composed of many Mg^2+^ dependent enzymatic reactions. In fact, dysregulation of Mg^2+^ homeostasis is involved in various cellular malfunctions and diseases. Recently, mitochondria, energy-producing organelles, have been known as major intracellular Mg^2+^ stores. Several biological stimuli alter mitochondrial Mg^2+^ concentration by intracellular redistribution. However, in living cells, whether mitochondrial Mg^2+^ alteration affect cellular energy metabolism remains unclear. Mg^2+^ transporter of mitochondrial inner membrane MRS2 is an essential component of mitochondrial Mg^2+^ uptake system. Here, we comprehensively analyzed intracellular Mg^2+^ levels and energy metabolism in *Mrs2* knockdown (KD) cells using fluorescence imaging and metabolome analysis. Dysregulation of mitochondrial Mg^2+^ homeostasis disrupted ATP production *via* shift of mitochondrial energy metabolism and morphology. Moreover, *Mrs2* KD sensitized cellular tolerance against cellular stress. These results indicate regulation of mitochondrial Mg^2+^ *via* MRS2 critically decides cellular energy status and cell vulnerability *via* regulation of mitochondrial Mg^2+^ level in response to physiological stimuli.

Adenosine triphosphate (ATP) is the universal energy currency of cells. ATP binds to magnesium ion (Mg^2+^) to compose biologically functional form, and most of intracellular ATP and Mg^2+^ assumed to form Mg-ATP complexes. Because both ATP and Mg^2+^ are mutually and strongly buffered in cytosol, it had been believed that Mg^2+^ deeply contributes on energy metabolism. Actually, studies performed *in vitro* has revealed that the variety of enzymatic activities are dependent on [Mg^2+^]^1^, and some of the Mg^2+^-dependent enzymes are operated in mitochondria to sustain the cellular function and viability. Some of enzymatic activities of tricarboxylic acid cycle (TCA cycle)^2^,^3^ are regulated by [Mg^2+^], and therefore, computer simulation by a realistic metabolic model of TCA cycle also showed mitochondrial Mg^2+^ level is most important regulating factor^4^. Mg^2+^ homeostasis is crucial for maintenance of electron transport chain^5^. Moreover, the mitochondrial ATP-Mg/P_i_ carrier exports mitochondrial ATP into cytosol^6^,^7^. Therefore, Mg^2+^ has been implicated as an important regulator of metabolic status in mitochondria^8^,^9^,^10^.

Mg^2+^ is an important cation for maintain cellular functions and, therefore, suggested the relation of Mg^2+^ to various diseases such as cancer, obesity, type 2 diabetes and neurological diseases^11^,^12^,^13^,^14^. Furthermore, intracellular Mg^2+^ plays roles as a second messenger in the immune system^15^,^16^,^17^,^18^, and it has been recognized as a multi-target metabolic regulator^8^,^19^. Therefore, regulation of intracellular Mg^2+^ is critical for maintenance of cellular functions and tissue integrity. To reveal the regulatory mechanism of intracellular Mg^2+^, we have developed Mg^2+^ sensitive fluorescence probes and imaging techniques^20^,^21^,^22^,^23^,^24^. These intracellular Mg^2+^ imaging works revealed Mg^2+^ mobilization in pathological and physiological conditions^25^,^26^,^27^,^28^, and mitochondria are intracellular Mg^2+^ stores^29^. Mitochondria redistribute cytosolic and mitochondrial Mg^2+^ sufficient to change the cytosolic Mg^2+^ concentration ([Mg^2+^]_cyto_) in response to several physiological stimuli^25^,^26^. Recent studies using novel Mg^2+^ fluorescent probe successfully visualized that mitochondrial Mg^2+^ concentration ([Mg^2+^]_mito_) dynamically changes^22^,^30^. However, it is not clear, in cells, how the changes of [Mg^2+^]_mito_ comprehensively affect the cellular energy metabolism in detail.

Although regulation of [Mg^2+^]_mito_ has not been elucidated in detail, mitochondrial Mg^2+^ channel MRS2 is known to be a molecular machinery associated with Mg^2+^ influx into mitochondria^31^,^32^,^33^,^34^. The rats with functional inactivation of mutated MRS2 have major mitochondrial deficits with a reduction in ATP, and increased numbers of mitochondria in oligodendrocytes^35^. Mg^2+^ uptake into mitochondria *via* MRS2 is essential for the maintenance of respiratory chain and cell viability^5^.

In this study, we investigate how dysregulation of mitochondrial Mg^2+^ homeostasis affects cellular energy maintenance and viability using single-cell fluorescence imaging and metabolomics analysis in *Mrs2* knockdown (KD) cells. *Mrs2* KD induces disruption of mitochondrial Mg^2+^ homeostasis, which results in supression of mitochondrial ATP production and increased cellular stress susceptibility. These findings suggest that mitochondrial Mg^2+^ plays important roles to maintain energy supply in cells, and its dysregulation causes cellular malfunction and multiple diseases.

## Results

### RNAi-mediated *Mrs2* KD in HeLa cells

We investigated the importance of mitochondrial Mg^2+^ homeostasis by an RNAi-mediated *Mrs2* KD in HeLa cells. The best miRNA to knockdown MRS2 expression was selected by comparing loss of *Mrs2* mRNA expression in HeLa cells with quantitative real time RT-PCR after 3 days of transfection of miR expression vector (Fig. S1). The miR expression vector #1 was optimal for *Mrs2* KD in HeLa cells, and it was used for *Mrs2* KD.

### Effects of *Mrs2* KD on Intracellular Mg^2+^ Homeostasis

**Figure 1:**
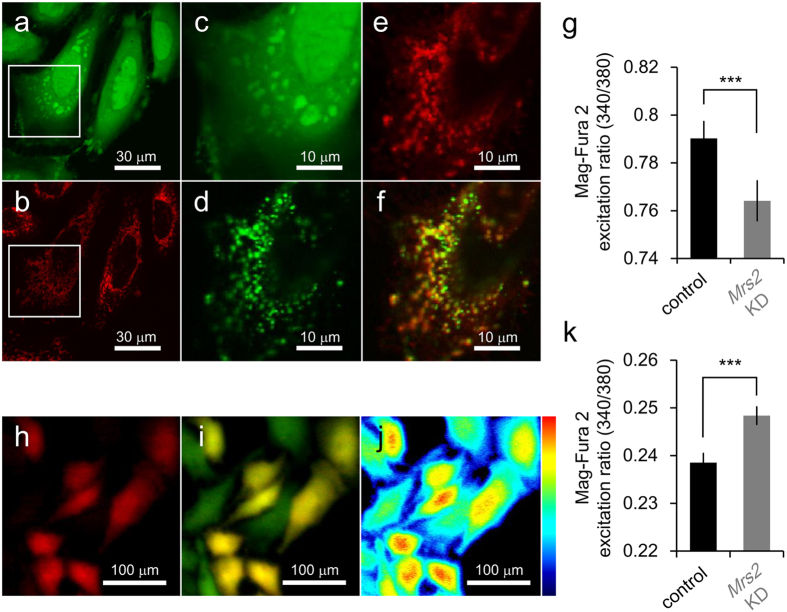
The effects of *Mrs2* KD on the intracellular Mg^2+^ homeostasis. The images of HeLa cells simultaneously loaded with both Mag-Fura-2 (**a**) and MitoFluor Red (**b**). (**c**–**f**) The enlarged view of the boxed area in panel a and b. (**c**,**d**) The localization of Mag-Fura-2 in the same cell shown in the panel a and b before (**c**) and after (**d**) cell membrane permeabilization by digitonin. (**e**) Localization of MitoFluor Red after cell membrane permeabilization by digitonin. (**f**) The merged image were shown. (**g**) The mitochondrial Mg^2+^ level in control and *Mrs2* KD HeLa cells. Comparing the ratio of Mag-Fura-2 fluorescence excited at 340 nm to at 380 nm, indicating mitochondrial Mg^2+^ level, revealed a decrease in *Mrs2* KD cells (n = 148 for the control and n = 105 for *Mrs2* KD cells from 5 experiments). (**h**–**j**) The images of HeLa cells normally loaded with Mag-Fura-2. (**h**) Fluorescence image of *Mrs2* KD cells labeled with emGFP. (**i**) The merged fluorescence images of control and emGFP- labeled (red) Mrs2 KD cells loaded with Mag-Fura-2 (green). (**j**) The fluorescence emission ratio image at excitation of 340 and 380 nm of HeLa cells loaded with Mag-Fura-2. Pseudo color showed the emission ratio of Mag-Fura-2 signals excited at 340 nm to 380 nm. (**k**) The cytosolic Mg^2+^ level in control and *Mrs2* KD cells. In *Mrs2* KD cells, cytosolic Mg^2+^ level is higher than that in control cells (n = 304 for the control and n = 384 *Mrs2* KD cells from 5 experiments).

MRS2 is primary Mg^2+^ uptake machinery in mitochondria^31^,^32^,^34^. To assess the effects of *Mrs2* KD on [Mg^2+^]_mito_ homeostasis, [Mg^2+^]_mito_ was compared by using a ratiometric Mg^2+^ indicator Mag-Fura-2. The cell membrane permeabilization protocol was used for the quantification of the [Mg^2+^]_mito_^36^. Briefly, after loading Mag-Fura-2, cytosolic Mag-Fura-2 was washed out by cell membrane permeabilization with a detergent digitonin. While Mag-Fura-2 is normally used for the measurement of [Mg^2+^]_cyto_ (Fig. 1a–c), the co-localization of Mag-Fura-2 and a mitochondrial marker MitoFluor Red signals was observed after Mag-Fura-2 wash out from cytosol by digitonin treatment (Fig. 1d–f), indicating that this cell membrane permeabilization protocol by using Mag-Fura-2 enables mitochondrial Mg^2+^ measurement. [Mg^2+^]_mito_ in *Mrs2* KD cells was lower than that in normal cells (Fig. 1g). *Mrs2* KD cells were identified by expression of emGFP also coded in miRNA expression vector. [Mg^2+^]_cyto_ was also estimated with Mag-Fura-2 in normal usage (Fig. 1h–j). The comparative analysis revealed the *Mrs2* KD induced an increase in [Mg^2+^]_cyto_ (Fig. 1k). These results indicate that *Mrs2* KD in HeLa cells induces a decrease in [Mg^2+^]_mito_ and an increase [Mg^2+^]_cyto_.

### Effects of *Mrs2* KD on Mitochondrial Mg^2+^ Regulatory Systems

**Figure 2:**
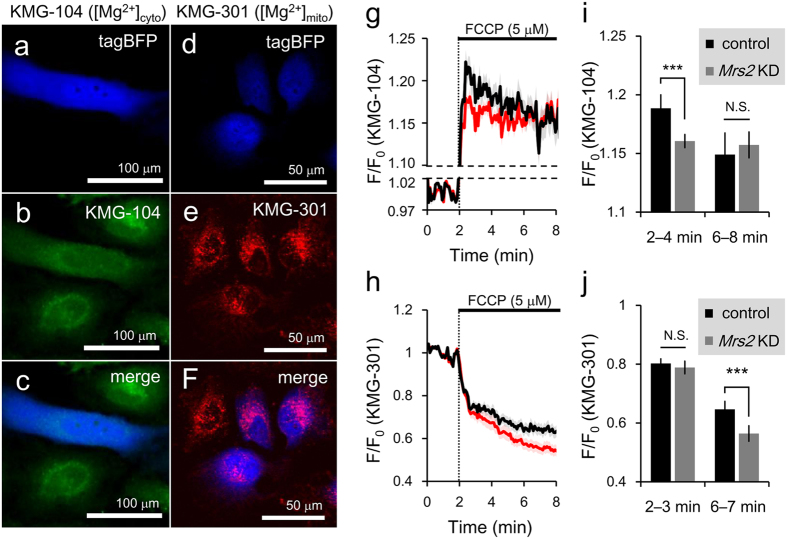
The effects of *Mrs2* KD on mitochondrial Mg^2+^ regulatory system. (**a**–**c**) Representative fluorescence image of control and *Mrs2* KD cells loaded with cytosolic Mg^2+^ indicator KMG-104. (**a**) *Mrs2* KD cells labeled with tagBFP. (**b**) Control and *Mrs2* KD cells stained with KMG-104. (**c**) The merged image of tagBFP and KMG-104 images. (**d**–**f**) Representative fluorescent image of control and *Mrs2* KD cells loaded with mitochondrial Mg^2+^ indicator KMG-301. (**d**) *Mrs2* KD cells labeled with tag BFP. (**e**) Control and Mrs2 KD cells stained with KMG-301. (**f**) The merged image of tagBFP and KMG-301 images. (**g**) Averaged time course of [Mg^2+^]_cyto_ dynamics in control and *Mrs2* KD cells measured with KMG-104. Treatment with mitochondrial uncoupler FCCP (5 μM) triggered the [Mg^2+^]_cyto_ increase (means ± SEM; black line n = 46 for control cells; red line n = 43 for *Mrs2* KD cells from 4 experiments). (**h**) Averaged time course of [Mg^2+^]_mito_ dynamics in control and *Mrs2* KD cells measured with KMG-301. Treatment with FCCP (5 μM) triggered the [Mg^2+^]_mito_ decrease in mitochondria (means ± SEM; black line, n = 46 for control; red line for n = 43 Mrs2 KD cells from 4 experiments). (**i**) Comparison of averaged amplitudes of [Mg^2+^]_cyto_ increase between control and *Mrs2* KD cells at 2–4 min (initial [Mg^2+^]_cyto_ increase, left) and 6–8 min (late phase, right). Data are represented as mean ± SEM (n = 46 for control and n = 43 for *Mrs2* KD cells from 4 experiments). (**j**) Comparison of averaged amplitudes of [Mg^2+^]_mito_ decrease between control and *Mrs2* KD cells at 2–3 min (initial [Mg^2+^]_mito_ decrease) and 6–7 min (after mitochondrial re-uptake of Mg^2+^ released into cytosol). Data are represented as means ± SEM (n = 46 for control and n = 43 *Mrs2* KD cells from 4 experiments).

The alternation in intracellular distribution of Mg^2+^ level in *Mrs2* KD cells indicates that MRS2 maintain mitochondrial Mg^2+^ concentration. To investigate the roles of MRS2 on stimulation-induced intracellular Mg^2+^ redistribution, we visualized cytosolic and mitochondrial Mg^2+^ dynamics during stimulation with a mitochondrial uncoupler, carbonyl cyanide p-(trifluoromethoxy) phenylhydrazone (FCCP; 5 μM) in HeLa cells loaded with highly sensitive cytosolic Mg^2+^ indicator KMG-104 (Fig. 2b,c) or mitochondrial Mg^2+^ indicator KMG-301 (Fig. 2e,f). To distinguish *Mrs2* KD cells from control cells, *Mrs2* miR expression vector coding tagBFP was used. As both control and tagBFP-labeled *Mrs2* KD cells existed in an observed area of single experiments, the traces of [Mg^2+^]_cyto_ and [Mg^2+^]_mito_ in both control and *Mrs2* KD cells for comparative analysis were simultaneously obtained in the same batch experiments (Fig. 2a–f). As shown in previous studies reporting FCCP-induced Mg^2+^ release from mitochondria into the cytosol^22^,^25^,^26^,^29^, the application of FCCP induced [Mg^2+^]_cyto_ increase (Fig. 2g). Measurement of [Mg^2+^]_cyto_ dynamics using KMG-104 revealed that the averaged amplitude of FCCP-induced [Mg^2+^]_cyto_ increase in 2–4 min (initial phase of [Mg^2+^]_cyto_ increase) in *Mrs2* KD cells was smaller than that in control cells (Fig. 2i, left), supporting the idea that the amount of Mg^2+^ stored in mitochondria before FCCP treatment is lower in *Mrs2* KD cells. The [Mg^2+^]_cyto_ increase was sustained at least for 8 min in *Mrs2* KD cells, whereas [Mg^2+^]_cyto_ in control cells returned back to the same level of [Mg^2+^]_cyto_ in *Mrs2* KD cells in 6–8 min (Fig. 2i, right). In [Mg^2+^]_mito_ imaging using KMG-301 (Fig. 2h), in 2–3 min (initial phase of [Mg^2+^]_mito_ decrease), the averaged amplitudes of FCCP-induced [Mg^2+^]_mito_ decrease in both cells were not different (Fig. 2j, left). During 6–7 min, the averaged amplitudes of FCCP-induced [Mg^2+^]_mito_ decrease in *Mrs2* KD cells was larger than that in control cells (Fig. 2j, right). These results also indicate that, after the initial phase of FCCP-induced mitochondrial Mg^2+^ release, mitochondria reuptake the released cytosolic Mg^2+^ through MRS2, and mitochondria in *Mrs2* KD cells failed it. Taken together, MRS2 plays a role as the mitochondrial Mg^2+^ uptake systems in mammalian cells, and *Mrs2* KD causes the deletion of mitochondrial Mg^2+^ uptake in both steady state and Mg^2+^ mobilization.

### Suppression of TCA Cycle Induced by Disruption of Mitochondrial Mg^2+^ Homeostasis

**Figure 3:**
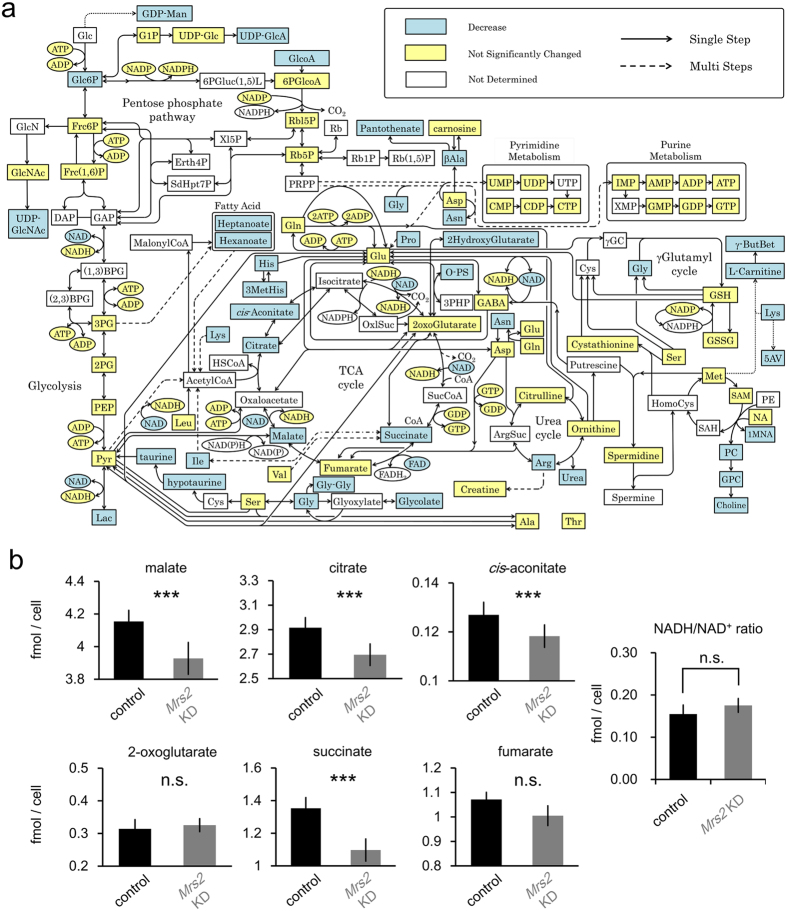
Metabolic alteration of *Mrs2* KD cells. (**a**) The Overview of alternation in metabolome between control and *Mrs2* KD cells. Significance was determined by paired *t*-test (n = 6, p < 0.05). The significantly decreased metabolites in *Mrs2* KD samples are shown in light blue boxes. All significantly changed metabolites are shown. Yellow Boxes indicate metabolites not significantly changed. (**b**) Comparison of metabolites in TCA cycles and NADH/NAD^+^ ratio comparing control and *Mrs2* KD cells. Significance was determined by paired *t*-test (n = 6, p < 0.05).

To directly assess the effect of *Mrs2* KD-induced homeostatic malfunction of mitochondrial Mg^2+^ on global metabolism and, in particular, mitochondrial energy generation, the metabolomics of *Mrs2* KD and control cells were investigated using capillary electrophoresis mass spectrometry (CE-MS) technique. CE-MS comprehensively quantify metabolites in biological samples^37^. We examined the differences in metabolite levels between control and *Mrs2* KD cells. We quantitatively identified a total of 133 metabolites (control and *Mrs2* KD samples, n = 6 respectively), and 24 metabolites were found to significantly differ between control and *Mrs2* KD samples, which are overviewed in Fig. 3a (pathway information was obtained by reference to KEGG [http://www.genome.jp/kegg/pathway.html] and a previous report^38^). Especially, most metabolites of TCA cycle such as malate, citrate, cis-aconitate and succinate, were reduced in *Mrs2* KD cells (Fig. 3b). These results suggest that dysregulation of mitochondrial Mg^2+^ homeostasis causes suppression of TCA cycle turnover in mitochondria. Despite suppression of TCA cycle activity, NADH/NAD^+^ was not affected in *Mrs2* KD cells (Fig. 3b). No effect on product of TCA cycle suggests that the direct impact of *Mrs2* KD is downstream of TCA cycle in mitochondrial energy production.

### *Mrs2* KD Disrupt the Mitochondrial Membrane Potential

**Figure 4:**
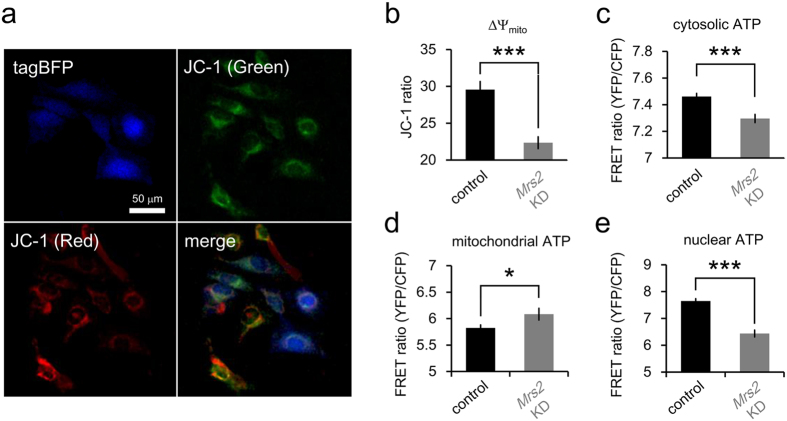
Comparison of ΔΨ and intracellular ATP level between control and *Mrs2* KD cells. (**a**) Representative fluorescent image of control and *Mrs2* KD cells stained with ΔΨ indicator JC-1 (top right: green fluorescence; bottom left: red fluorescence; bottom right: merged image). *Mrs2* KD cells were labeled with tagBFP (top left). (**b**) Comparative analysis of the green-to-red ratio of JC-1 signals (index of ΔΨ) revealed that *Mrs2* KD decreases the ΔΨ (n = 955 and for control and n = 375 *Mrs2* KD cells from 4 experiments). (**c**–**e**) Cytosolic (**c**), mitochondrial (**d**) and nucleic (**e**) ATP levels in control and *Mrs2* KD cells were measured with genetically encoded ATP sensor ATeam. In *Mrs2* KD cells, ATP level was decreased in cytosol (n = 1944 control cells from 8 experiments and n = 556 *Mrs2* KD cells from 8 experiments) and nucleus (n = 621 control cells from 5 experiments and n = 119 *Mrs2* KD cells from 5 experiments), and increased in mitochondrial (n = 388 control cells from 4 experiments and n = 126 *Mrs2* KD cells from 4 experiments).

Using electron released from NADH and FADH_2_ produced in TCA cycle, proton gradient is generated across mitochondrial inner membrane *via* electron-transport chain, which result in generation of mitochondrial membrane potential (ΔΨ). To assess the ΔΨ, we quantified the ratio (red to green) of average mitochondrial fluorescence intensity of 5, 5′, 6, 6′-terachloro-1, 1′, 3, 3′-tetraethylbenzimidazolylcarbocyanine iodide (JC-1) per cells (Fig. 4a). In *Mrs2* KD HeLa cells, the red-to-green ratio of JC-1 fluorescence, which indicates the mitochondrial inner membrane potential, was lower than control cells (Fig. 4b). This result suggests the mitochondrial Mg^2+^ uptake through MRS2 is crucial for maintaining the ΔΨ.

### *Mrs2* KD Disrupt the Energy Metabolism in Mitochondria

In mitochondria, ATP is made from ADP and phosphate by ATP synthase using an electrochemical gradient of protons across the mitochondrial inner membrane. Therefore, inside-negative ΔΨ across the mitochondrial inner membrane is crucial for maintaining the physiological function of the oxidative phosphorylation to generate ATP. To access the intracellular energy status, we performed live imaging of cells expressing ATeam, which is genetically-encoded fluorescent resonance energy transfer (FRET)-based ATP indicator^39^. In cytosol and nucleus, the ATeam ratio, which indicates ATP level, were lower in *Mrs2* KD cells than that in control cells (Fig. 4c,e). In contrast to the decrease in extra-mitochondrial ATP levels, in *Mrs2* KD cells, ATP level increased in mitochondria (Fig. 4d) despite decrease of TCA cycle turnover (Fig. 3) and ΔΨ_m_ (Fig. 4a,b), suggesting that dysregulation of mitochondrial Mg^2+^ causes the imbalances of ATP exports from mitochondria.

### Dysregulation of Mitochondrial Mg^2+^ Affect the Mitochondrial Morphology

**Figure 5:**
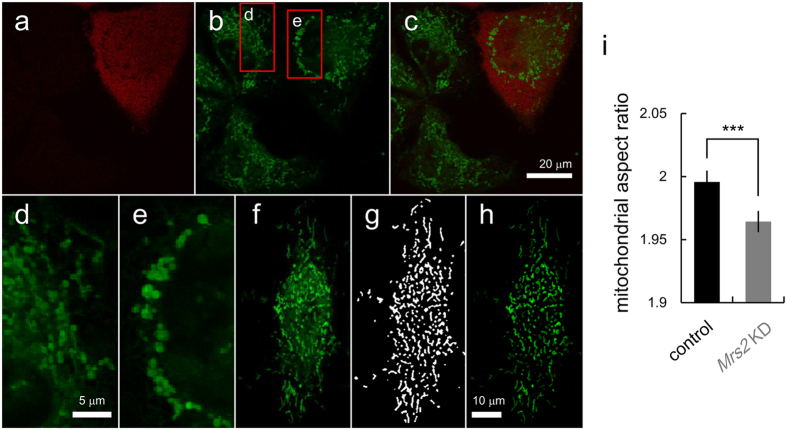
Morphological analysis of mitochondria in control and *Mrs2* KD cells. (**a**) *Mrs2* KD cells were labeled with tagBFP. (**b**) In control and *Mrs2* KD cells, mitochondrial location and shape were visualized with MitoTracker Green FM. (**c**) The merged image was shown (green: mitochondria, red: *Mrs2* KD cells). (**d**,**e**) The enlarged images of boxed area in panel b in control (**d**) and *Mrs2* KD cells (**e**). (**f**) Representative image of mitochondria stained for digital image processing. (**g**) Binary image of mitochondria shown in panel (**f**). (**h**) Fluorescent image of mitochondria masked with mitochondrial binary image shown in panel g. (**i**) Comparison of aspect ratio (AR) of mitochondrial shape between in control and in *Mrs2* KD cells. The AR of mitochondria in *Mrs2* KD cells was smaller than that in control cells, suggesting the rounding of mitochondria (mean ± SEM; n = 20628 mitochondria in 109 cells for control and n = 20159 mitochondria in 84 cells from 8 experiments).

Mitochondria are highly dynamic organelle^12^,^40^,^41^, and their morphological changes regulate cellular metabolic processes and *vice versa*^12^. We assessed whether the malfunction of mitochondrial Mg^2+^ regulatory system affects mitochondrial morphology. To obtain the exact mitochondrial morphology, mitochondrion selective probe insensitive to ΔΨ, Mito Tracker Green FM, was loaded into both control and *Mrs2* KD cells (Fig. 5b,c). To distinguish *Mrs2* KD cells from control cells, *Mrs2* miR expression vector coding tagBFP was used (Fig. 5a,c). Whereas the normal morphology of mitochondria in cells was tubular^10^ (Fig. 5d), the increased accumulation of rounding mitochondria was observed in *Mrs2* KD cells (Fig. 5e). In stress condition, the abnormal accumulation of large swollen mitochondria is reported in previous studies^42^,^43^. To quantitatively analyze the morphological difference of mitochondria between in control and in *Mrs2* KD cells, a morphological feature was evaluated by computer-assisted image processing. The acquired images (Fig. 5f) were processed to generate a mitochondria-specific binary image (Fig. 5g) allowing the quantification of mitochondrial shape (Fig. 5h) as previously described^44^. From quantitative morphological analysis of mitochondria, the aspect ratio (the ratio between the major and minor axes of the ellipse equivalent to the mitochondrial object) was calculated for each mitochondrion. The mitochondria in *Mrs2* KD cells had lower aspect ratio than that in control cells (Fig. 5i), indicating that *Mrs2* KD induced mitochondrial rounding.

### Cell Vulnerability to Cellular Stress enhanced by Mitochondrial Mg^2+^ dysregulation

**Figure 6:**
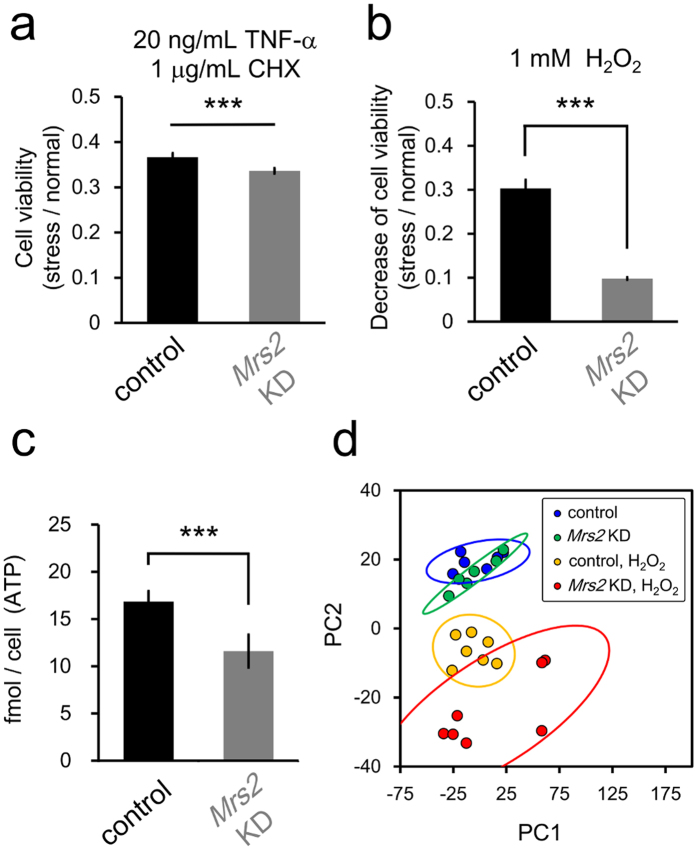
*Mrs2* KD induced increased vulnerability to cellular stress. (**a**,**b**) Comparison of the cell viability under TNF-α/CHX- or H_2_O_2_-induced stress between in control and in *Mrs2* KD cells. In *Mrs2* KD cells, cell damage (decrease rate in cell viability) in the presence of H_2_O_2_ (**a**) or TNF-α/CHX (**b**) is more severe than that in control cells (n = 12 for the control and n = 12 for *Mrs2* KD samples), indicating *Mrs2* KD cells are more vulnerable to cellular stress. (**c**) Comparison of ATP level between control and *Mrs2* KD cells under H_2_O_2_ condition by metabolome analysis (n = 6 for each sample). (**d**) Plot of samples under 4 conditions projected onto the first two principal components (PC1 and PC2) identified PCA using all quantified 104 metabolites (n = 6 or 7 for each sample). Each circle indicates a single sample, while the larger ovals represent 95% confidence regions. Each color indicates 4 groups of conditions.

We revealed the dysregulation of mitochondrial Mg^2+^ results in the reduction of extra-mitochondrial ATP concentration. The negative effects of low ATP levels on cellular vulnerability are suggested^45^. To investigate the physiological effects of inaccessibility to Mg^2+^ induced energy imbalance on cellular vulnerability to stress, the viabilities were compared under cellular stress conditions between control and *Mrs2* KD cells by MTT assay. In *Mrs2* KD cells, cell viabilities were lower than in control cells in the condition of 20 ng/mL TNF-α and 1 μg/mL cycloheximide (CHX) (Fig. 6a) and also 1 mM H_2_O_2_ conditions (Fig. 6b), respectively. These results indicate that dysregulation of mitochondrial Mg^2+^ homeostasis causes increased susceptibility under cellular stress conditions. Metabolome analysis revealed that, under H_2_O_2_ condition, ATP level in *Mrs2* KD cells was lower than that in control cells (Fig. 6c), suggesting *Mrs2* KD-induced lower ATP level cause cellular vulnerability against oxidative stress. Next, to investigate the effects of mitochondrial Mg^2+^ dysregulation on cellular metabolome under stress condition, the overall impact of *Mrs2* KD was compared using principal component analysis (PCA) for all detected 104 metabolites among 4 conditioned samples; control and *Mrs2* KD cells with/without H_2_O_2_. PCA revealed that the clusters of control and *Mrs2* KD cells under H_2_O_2_ condition were more separated than in normal condition, indicating that *Mrs2* KD-induced defection of mitochondrial ATP synthesis had larger impacts under stress conditions (Fig. 6d).

## Discussion

We demonstrated that deficit of MRS2, which is a mitochondrial primary Mg^2+^ regulatory system, causes disruption of mitochondrial energy metabolism and cellular sensitization against cellular stress. First, we confirmed that *Mrs2* KD causes malfunction of mitochondrial Mg^2+^ uptake (Fig. 2) and reduction of Mg^2+^ stored in mitochondria (Fig. 1). Second, we revealed that *Mrs2* KD induces decreases in substrates of TCA cycle (Fig. 3), ΔΨ and extra-mitochondrial ATP levels (Fig. 4) in contrast increased mitochondrial ATP level (Fig. 4). In addition, we observed that mitochondria in *Mrs2* KD cells have abnormal morphology (Fig. 5). Lastly, we showed that the effect of *Mrs2* KD was noticeable under stress conditions, which sensitized cells to cellular stress (Fig. 6). These results indicate that mitochondrial Mg^2+^ regulate the cellular energy status, and it changes mitochondrial morphology and affects cell vulnerability against biological stress.

### Physiological Significance of mitochondrial Mg^2+^ homeostasis

Although partial information has been accumulated about Mg^2+^-dependent regulation of energy metabolism, the comprehensive effects of mitochondrial Mg^2+^ on metabolic status in living cells have not been elucidated. As far as we know, this is the first work to demonstrate a series of relationships between Mg^2+^ regulatory system and cellular energy metabolism. In *Mrs2* KD cells, in contrast to decreased substrates of TCA cycle (Fig. 3), collapse of ΔΨ_m_ and decreased extra-mitochondrial ATP levels (Fig. 4), mitochondrial ATP level are increased (Fig. 4). These results indicate the mitochondrial dis-accessibility to Mg^2+^ suppress ATP efflux from mitochondria, which is possibly mediated by ATP-Mg/P_i_ carrier^6^. Mitochondrial ATP accumulation inhibits many enzymatic processes in TCA cycle^46^ and electron transport chain activities^5^ in a negative feedback manner. Suppression of TCA cycle and electron transport chain activities would result in reduced ATP production in mitochondria. Consequently, mitochondrial Mg^2+^ regulates coupled reactions in mitochondrial energy metabolism, i.e. TCA cycle, electron transport chain and ADP/ATP translocation. In addition, morphological changes of mitochondria were also observed in *Mrs2* KD cells (Fig. 5). Abnormal large and round mitochondria are also observed under pharmaceutically ATP synthesis-inhibited condition^47^. These are consistent with the idea that mitochondrial morphology is controlled by energy metabolism^48^. Abnormal mitochondrial morphology is associated with cancer^11^ obesity, type 2 diabetes^12^, and neurodegenerative disorders^12^,^13^,^14^. It may be explained by the idea that metabolic impairment induces cellular vulnerability^45^. Actually, sensitization against cellular stress by mitochondrial Mg^2+^ dysregulation was observed in *Mrs2* KD cells (Fig. 6). In contrast, in a cellular model experiments of Parkinson’s disease, increase in [Mg^2+^]_cyto_, which probably links to [Mg^2+^]_mito_ increase, protects cells from neurodegeneration by maintaining cellular ATP concentration and suppressing ROS production^28^. In summary, mitochondrial Mg^2+^ regulate the cellular metabolic process *via* shift of mitochondrial energy metabolism, and it changes mitochondrial morphology and affects the cell viability through changing stress susceptibility.

In normal cells, mitochondrial Mg^2+^ would play a role as a regulator of metabolic state under physiological condition. A wide variety of hormonal regulations of intracellular Mg^2+^ homeostasis has been reported^49^. In human, circadian rhythm for the serum Mg^2+^ level with the peak around noon are reported^50^, which is corresponding to circadian Mg^2+^ excretory rhythm with the peak at night^51^. In facts, a recent study revealed circadian rhythms in the intracellular [Mg^2+^] regulate cellular metabolism^52^. Because Mg^2+^ transport through MRS2 depends on extra-mitochondrial Mg^2+^ concentration^53^, mitochondrial Mg^2+^ homeostasis is also probably governed by circadian regulation. In addition, we recently demonstrated mitochondrial Mg^2+^ regulation mediated by second messenger pathways^26^. These studies support that cells dynamically regulate [Mg^2+^]_mito_ in response to various signals and circadian rhythm under physiological condition. A respiration-dependent mitochondrial Mg^2+^ regulation^54^ is consistent with the coupling of [Mg^2+^]_mito_ regulation with mitochondrial activities. Consequently, cells would modulate energy production and metabolic states *via* regulation of mitochondrial Mg^2+^ in response to environmental information.

## Methods

### HeLa cells culture

HeLa cells were seeded in Dulbecco’s modified Eagle’s medium (DMEM) supplemented with 10% heat-inactivated fetal bovine serum (FBS) and 50 U/mL penicillin and 50 mg/mL streptomycin (Life technologies, Carlsbad, CA, USA) and cultured at 37 °C in a humidified atmosphere containing 5% CO_2_. The cells were plated on glass-bottomed dishes (Iwaki, Tokyo, Japan), 100 mm cell culture dishes (Thermo Fisher Scientific, Waltham, MA, USA), or 48 well dishes (Thermo Fisher Scientific) for fluorescence imaging, metabolomics analysis or MTT assay, respectively. The medium was changed in every other day.

### Knockdown of *Mrs2* in HeLa cells

For knockdown of *Mrs2*, BLOCK-iT^TM^ Pol II miR RNAi Expression Vector Kits (Life technologies) was used. BLOCK-iT RNAi Designer from Life Technologies was used to design four single-stranded DNA oligonucleotides encoding the target pre-miRNA. These vectors are specifically designed to allow expression of miRNA sequences and contain specific miR flanking sequences that allow proper processing of the miRNA. Sequences of pre-miRNA insert in miRNA vectors for knockdown were designed as follow:

5′-GTAGCTACTGATTCCAAGAGTAGTTTTGGCCACTGACTGACTACTCTTGATCAGTAGCTA-3′ (*Mrs2* KD #1),

5′-GAATGCTAGCTACTGATTCCAAGTTTTGGCCACTGACTGACTTGGAATCTAGCTAGCATT-3′ (*Mrs2* KD #2),

5′-GTGAAGAATGCTAGCTACTGATGTTTTGGCCACTGACTGACATCAGTAGAGCATTCTTCA-3′ (*Mrs2* KD #3), and

5′-GTTTGAAGAATGCTAGCTACTGGTTTTGGCCACTGACTGACCAGTAGCTCATTCTTCAAA-3′ (*Mrs2* KD #4), respectively.

To identify the cells transfected with microRNA expression vector for *Mrs2* knockdown, the sequence coding EmGFP or tagBFP were incorporated into the vectors. The miR RNAi vectors were transfected into cells 3 days prior to the experiments using Lipofectamine LTX (Life technologies) in the experiments except preparation for metabolome analysis.

### Real time RT-PCR

To determine *Mrs2* mRNA levels in miR transfected cells, total RNA from HeLa cells were isolated and purified by using the RNeasy mini kit (QIAGEN, Tokyo, Japan). Total RNA was reverse-transcripted using SuperScript VILO (Life technologies), and the generated cDNA was used as template for quantitative real time PCR amplification with SYBR GreenER^TM^ (Life technologies). The *Mrs2* mRNA levels were normalized to GAPDH signals as an internal standard. Quantitative real time PCR were performed using the following primers: *Mrs2* forward 5′-CAGTTGTCTGGAGAGGGTCAA-3′, *Mrs2* reverse 5′-AAGGATCAGTGGCTGCAAAA-3′, GAPDH forward 5′-CACCCACTCCTCCACCTTTG-3′ and GAPDH reverse 5′-CATGAGGTCCACCACCCTGT-3′.

### Fluorescence imaging of the cytosolic or mitochondrial Mg^2+^ levels

For the quantification of cytosolic Mg^2+^ level in cells, HeLa cells were loaded with Mag-Fura-2-AM (Life technologies). For dye loading, HeLa cells were incubated in medium with 1 μM and 0.02% F-127 for 45 min at 37 °C in a humidified atmosphere containing 5% CO_2_. The cells were gently washed twice with 1.0 mL of Hanks’ balanced salt solution (HBSS) at pH7.4 (adjusted with NaOH) that consisted of (in mM) 137 NaCl, 5.4 KCl, 1.3 CaCl_2_, 0.5 MgCl_2_, 0.4 MgSO_4_, 0.3 Na_2_HPO_4_, 0.4 KH_2_PO_4_, 4.2 NaHCO_3_, 5.6 D-glucose, 5.0 HEPES. Then, further incubation was carried out for 15 min to allow for complete de-esterification of AM esters. For the measurement of the emitted fluorescence values at the excitation of 340 nm and 380 nm, a fluorescence microscope ECLIPSE TE300 (Nikon, Tokyo, Japan) equipped with a ×20 objective (S Fluor, Nikon) was used. A Xe lamp (150 W) with a monochromator unit was used for 340 nm and 380 nm excitation, and fluorescence was measured with a CCD camera (HiSCA, Hamamatsu Photonics, Shizuoka, Japan). The ratios of the fluorescence with excitation at 340 nm to that at 380 nm were calculated as an indicator of the cytosolic Mg^2+^ levels. For the detection of *Mrs2* KD cells, emGFP was illuminated with the excitation at 488 nm. Region of interest (ROI) was located on the respective cell areas. In each ROI, spatial averaged ratios of the emitted fluorescence with excitation at 340 nm to that at 380 nm was calculated as an indicator of the Mg^2+^ levels.

For the quantification of mitochondrial Mg^2+^ level in cells using ratiometric Mg^2+^ indicator Mag-Fura-2, the cell permeabilization method using digitonin was performed as described previously^36^. Briefly, cells were stained with Mag-Fura-2 as described above. Cells loaded with Mag-Fura-2 were permeabilized with 20 μg/mL digitonin in intracellular-like medium (ICM; 120 mM KCl, 10 mM NaCl, 1 mM KH_2_PO_4_, 20 mM HEPES-Tris at pH7.2 and 2 mM MgATP) buffer for 3 min, followed by washout of the released cytosolic Mag-Fura-2 with ICM. For the confirmation of mitochondrial localization and leakage from cytosol of Mag-Fura-2, fluorescence imaging was conducted with a confocal laser scanning microscope system (FluoViewFV1000; Olympus, Tokyo, Japan) mounted on an inverted microscope (IX81; Olympus) equipped with a ×60 oil objective. For the measurements Mag-Fura-2 signals, the cells were illuminated with the excitation wavelength at 405 nm from diode laser, and the signals from Mag-Fura-2 and Mito Fluor Red were separated using a 560 nm dichroic mirror. The fluorescence images were obtained by detecting the signals at 500–550 nm for Mag-Fura-2 and at 570–670 nm for Mito Fluor Red, respectively. For the measurement of the emitted fluorescence values at the excitation of 340 nm and 380 nm, a fluorescence microscope ECLIPSE TE300 equipped with a ×20 objective (S Fluor) was used. The procedure of Mag-Fura-2 detection was mentioned above. A ROI is located on the all spotted area with strong intensities in each cell (Fig. 1D).

### Real time imaging of cytosolic Mg^2+^ dynamics

For real time imaging of cytosolic Mg^2+^ dynamics, highly selective Mg^2+^ fluorescent dye KMG-104AM was used^24^. For loading KMG-104, cells were incubated with 5 μM KMG-104AM and 0.02% F-127 in pH adjusted HBSS for 30 min at 37 °C. Then, the cells were washed twice with HBSS and incubated in HBSS for 15 min at 37 °C in a humidified atmosphere containing 5% CO_2_ to allow for complete hydrolysis of the acetoxymethyl ester form. Fluorescence imaging was conducted with a confocal laser scanning microscope system, FV1000, equipped with a ×40 oil objective. For the measurements of KMG-104 signals, the cells loaded with KMG-104 were illuminated with the excitation wavelength at 488 nm from argon (Ar) laser. The fluorescence was obtained by detecting the signals at 500–600 nm. For the detection of tagBFP-labeled *Mrs2* KD cells, the cells were illuminated with the excitation wavelength at 405 nm from diode laser, and its fluorescence was obtained by detecting the signals at 425–475 nm.

### Real time imaging of mitochondrial Mg^2+^ dynamics

For real time imaging of mitochondrial Mg^2+^ dynamics, HeLa cells were stained with 20 μM highly selective mitochondrial Mg^2+^ fluorescent dye KMG-301AM in pH adjusted HBSS for 10 min on ice, so that hydrolysis of the acetoxymethyl ester by esterase present in the cytosol would be avoided^22^. Then, the cells were washed twice with HBSS and incubated in HBSS for 15 min at 37 °C in a humidified atmosphere containing 5% CO_2_ to allow for complete hydrolysis of the acetoxymethyl ester form in mitochondria. The detailed property and usage of KMG-301AM was described in our previous report^22^. Fluorescence imaging was conducted with a confocal laser scanning microscope, FV1000, equipped with a ×40 oil objective. The cells loaded with KMG-301 were illuminated with the excitation wavelength at 559 nm from diode laser, and its fluorescence was obtained by detecting signals at 570–670 nm. For the detection of tagBFP-labeled *Mrs2* KD cells, the cells were illuminated with the excitation wavelength at 405 nm from diode laser, and its fluorescence was obtained by detecting the signals at 425–475 nm.

### Fluorescence imaging of the intracellular ATP levels

For the quantification of intracellular ATP levels in living cells, ATeam1.03^39^ was transfected into HeLa cells using Lipofectamine LTX. The transfection was conducted the day before observation. Fluorescent imaging was conducted with a confocal laser scanning microscope system equipped with a ×20 air objective. For the measurements of ATeam signals, the cells were illuminated with the excitation wavelength at 440 nm from diode laser, and the signals from mseCFP and cp173-mVenus were separated using a 510 nm dichroic mirror and obtained at 480–510 nm for mseCFP and at 535–565 nm for cp173-Venus, respectively. The ratios of cp173-mVenus to mseCFP signals were calculated as an indicator of the cytosolic ATP levels.

### Fluorescence imaging of the mitochondrial membrane potentials

For the quantification of mitochondrial membrane potential in living cells, HeLa cells were loaded with mitochondrial membrane potential sensitive dye 5, 5′, 6, 6′-terachloro-1, 1′, 3, 3′-tetraethylbenzimidazolylcarbocyanine iodide (JC-1; Life technologies). For dye loading, HeLa cells were incubated in pH adjusted HBSS with 10 μg/mL JC-1 for 15 min at 37 °C in a humidified atmosphere containing 5% CO_2_. The cells were gently washed twice with 1.0 mL of HBSS. Fluorescent imaging was conducted with a confocal laser scanning microscope system, FV1000, equipped with a ×20 air objective. For the measurements of JC-1 signals, the cells were illuminated with the excitation wavelength at 488 nm from Ar laser, and the signals from JC-1 were separated using a 560 nm dichroic mirror and obtained by detecting the signals at 520–560 nm for green channel and at 575–620 nm for red channel, respectively. The ratio of signals from red channels to that from green channels was calculated as an indicator of the mitochondrial membrane potential. For the detection of tagBFP-labeled *Mrs2* KD cells, the cells were illuminated with the excitation wavelength at 405 nm from diode laser, and its fluorescence images were obtained by detecting the signals at 425–475 nm.

### Measurement of mitochondrial morphology

For assessment of mitochondrial morphology in living cells, mitochondria were stained with Mito Tracker Green FM and their morphology were quantified by handmade digital image processing software using MATLAB (MathWorks, Cambridge, UK). The algorithm for quantification of morphological feature is previously described^44^. Briefly, mitochondrial binary images were obtained from Mito Tracker Green FM-stained images, and aspect ratio of each mitochondrion was calculated as the ratio between the major and minor axes of the ellipse equivalent to the mitochondrial object.

### Measurement of cell viability

For quantification of vulnerability against cellular stress, cell viabilities under the stress inducer H_2_O_2_- or TNFα/CHX-treated condition were measured using MTT assay. Control and *Mrs2* KD cells were treated with H_2_O_2_ (1 mM) or TNFα (20 ng/mL) plus cycloheximide (CHX; 1 μg/mL) for 24 h. Then, the cells were incubated in the medium containing 0.5 mg/mL of MTT for 2 h at 37 °C in a humidified atmosphere containing 5% CO_2_. Then, the medium was removed, and 100 μL of dimethylsulfoxide (DMSO, nacalai tesque, Kyoto, Japan) was added in each well to dissolve the precipitation. The absorbance at 570 nm under stress condition (ABS_stress_) and normal condition (ABS_control_) were measured using a microplate reader (Fluoroskan Ascent FL, Thermo Fisher Scientific). Cell viability was defined as the ABS_stress_/ABS_control_.

### Image analysis and statistics

The fluorescence was calculated as the mean intensity over a ROI on the cell body of each cell using the software package, FluoView (Olympus), Aquacosmos (Hamamatsu Photonics) or handmade software by MATLAB.

### Metabolome analysis

The cells were transfected with a plasmid for *Mrs2* knockdown by electroporation using Neon (Life Technologies). The cells were plated on a 100 mm dish, and grown for 3 days at 37 °C in a humidified atmosphere containing 5% CO_2_. Culture medium was changed every day. Procedure for sample preparation of metabolome analysis was previously described^37^. Briefly, after washing cells twice with ice-cold 5% mannitol, metabolites were extracted by 1 mL ice-cold methanol containing internal standards (25 μM each of methionine sulfone (MetSul; Wako, Osaka, Japan), 2-(N-morpholino)ethanesulfonic acid (MES; wako), D-Camphor-10-sulfonic acid (CSA; Wako). 400 μL of collected extracts were transferred into another tube, mixed with 400 μL chloroform and 200 μL Milli-Q water, and centrifuged at 10,000 × g for 3 min at 4 °C. A 400 μL aliquot of the aqueous layer was centrifugally filtered through a 5 kDa cutoff membrane (UltrafreeMC-PLHCC for Metabolome Analysis; Human Metabolome Technologies, Yamagata, Japan) to remove proteins from samples, followed by the centrifugal-concentration at 42 °C. CE-MS experiments were performed using Agilent CE Capillary Electrophoresis System.

## Additional Information

**How to cite this article**: Yamanaka, R. *et al*. Mitochondrial Mg^2+^ homeostasis decides cellular energy metabolism and vulnerability to stress. *Sci. Rep.* **6**, 30027; doi: 10.1038/srep30027 (2016).

## Supplementary Material

Supplementary Information
