# Supplementary material for: Mitochondrial Mg2+ homeostasis decides cellular energy metabolism and vulnerability to stress
Source: Sci Rep. 2016 Jul 26;6:30027. doi: 10.1038/srep30027 (PMC4960558; doi:10.1038/srep30027)
Supplement: Supplementary Information [file srep30027-s1.pdf]

Supplementary Information

Mitochondrial Mg^{2+} homeostasis decides Cellular energy metabolism and vulnerability to stress

Ryu Yamanaka, Sho Tabata, Yutaka Shindo, Kohji Hotta, Koji Suzuki, Tomoyoshi Soga and Kotaro Oka*

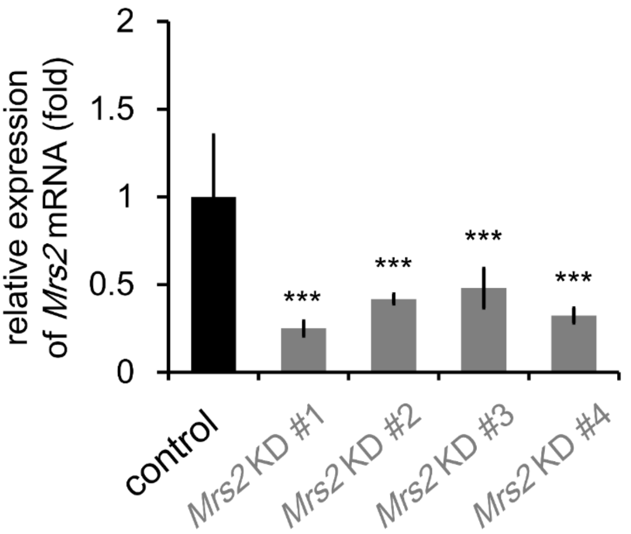

Figure. S1.

Relative expression levels of Mrs2 mRNA in HeLa cells transfected with expression vectors for miR RNAi against Mrs2 (Mrs2 RNAi #1, #2, #3 and #4) were estimated with real-time PCR. In cells transfected with the miR RNAi against Mrs2, the expression levels of *Mrs2* mRNA were suppressed. Relative Mrs2 mRNA expression levels were determined using glyceraldehyde-3-phosphate dehydrogenase (GAPDH) as an internal standard, and each expression level was normalized with that of mock-transfected cells. Expression levels were measured in 3–4 different samples and compared with that in mock-transfected cells. The error bars indicate SEM. *** indicates $p < 0.05$ (Dunnett's test).
